# A novel cuproptosis-related prognostic signature and potential value in HCC immunotherapy

**DOI:** 10.3389/fmolb.2022.1001788

**Published:** 2022-09-29

**Authors:** Xiang-Xu Wang, Li-Hong Wu, Hongchen Ji, Qing-Qing Liu, Shi-Zhou Deng, Qiong-Yi Dou, Liping Ai, Wei Pan, Hong-Mei Zhang

**Affiliations:** ^1^ Department of Clinical Oncology, Xijing Hospital, Fourth Military Medical University, Xi’an, Shaanxi, China; ^2^ Xijing 986 Hospital Department, Fourth Military Medical University, Xi’an, Shaanxi, China

**Keywords:** hepatocellular carcinoma, cuproptosis, prognostic signature, immunotherapy, cancer subtype

## Abstract

**Background:** Copper metabolism plays an important role in the tumor microenvironment, and cuproptosis is the last discovered programmed cell death process. However, the potential mechanism of cuproptosis in regulating the immune microenvironment of HCC remains unclear.

**Methods:** A total of 716 HCC patients with complete mRNA expression and survival information were collected from three public HCC cohorts (TCGA-LIHC cohort, *n* = 370; GSE76427 cohort, *n* = 115; ICGC-LIRI cohort, *n* = 231). The unsupervised clustering analysis (NMF) was performed to identify three different cuproptosis-related subtypes. The univariate-Cox, lasso-Cox and multivariate-Cox regression analyses were performed to screen the cuproptosis related and construct the cuproptosis-related prognosis signature (Cu-PS). The immune cell infiltration was estimated by both CIBERSORT and MCPcounter algorithms.

**Results:** This study identified three distinct cuproptosis-related metabolic patterns, which presented different pathway enrichment and immune cell infiltration. The Cu-PS, a 5-genes (C7, MAGEA6, HK2, CYP26B1 and EPO) signature, was significantly associated with TNM stage, tumor mutational burden (TMB), drugs sensitivity, and immunotherapies response.

**Conclusion:** This study performed a multi-genetic analysis of cuproptosis-related genes and further explored the regulatory mechanism of cuproptosis in HCC. The Cu-PS might be a useful biomarker for predicting immunotherapy response and enhancing the diagnosis and treatment of HCC.

## Introduction

Hepatocellular carcinoma (HCC) has high heterogeneity and poor prognosis (Dong et al., 2020) and led to the second-highest mortality rate among all cancers (Sung et al., 2021). The 5-year survival rate of HCC patients is less than 20% despite the continuous emergence of immunotherapy and targeted drugs (Llovet et al., 2021). Due to its high heterogeneity, the prognosis of HCC also varies greatly (Pinero et al., 2020). Alpha-fetoprotein (AFP), AFP-L3, glypican-3 (GPC3) and des-gamma-carboxy prothrombin (DCP) are important markers for the diagnosis and prognostication of HCC, but the sensitivity and specificity are limited (Cai et al., 2019; Choi et al., 2019). Therefore, finding novel regulatory mechanisms of HCC and identifying more reliable prognostic markers are crucial for improving the survival rate and promoting precision therapy.

Programmed cell death (PCD) is a process in which cells die after being stimulated by an external signal, which may be physiological or pathological (Hotchkiss et al., 2009). Apoptosis, autophagy and necrosis are three major forms of PCD that have been extensively studied (D'Arcy, 2019). In recent years, new PCD patterns including ferroptosis, pyroptosis, and necroptosis have been discovered, which play an important role in the occurrence and development of tumors (Frank and Vince, 2019; Hirschhorn and Stockwell, 2019; Tsvetkov et al., 2022). Copper ionophore-induced cell death, also called cuproptosis, is the latest proposed PCD pattern. The main mechanism is that copper binds to lipoylated components in the TCA cycle under overload conditions, thus inducing cell death (Tsvetkov et al., 2022). Copper metabolism plays an important role in the regulation of tumor microenvironment, however, the underlying mechanism of cuproptosis in HCC remains unclear. Therefore, it is crucially important to explore the association between cuproptosis and HCC, and to find new strategies for diagnosis and treatment.

In this study, we first preformed multi-genetic analysis of cuproptosis-related genes and further identified three cuproptosis-subgroups by nonnegative matrix factorization (NMF) clustering analysis. Differential pathway enrichment and immune cell infiltration analyses of these three subgroups were conducted to explore the biological mechanisms underlying the three subtypes. The cuproptosis-related prognostic signature (Cu-PS) was established based on the five identified prognostic core genes. The Cu-PS was significantly associated with TNM stage, tumor mutational burden (TMB), drug sensitivity, and immunotherapy response. In conclusion, our study explored the regulatory mechanism of cuproptosis in HCC and provides new targets and strategies for the clinical diagnosis and treatment of HCC patients.

## Materials and methods

### Data collection and preprocessing

A total of 716 HCC samples with complete survival and mRNA expression information from three HCC cohorts (TCGA-LIHC cohort, *n* = 370; GSE76427 cohort, *n* = 115; ICGC-LIRI cohort, *n* = 231) were included in this study. The clinical information and copy number variation data were downloaded from https://www.cancer.gov/. The mRNA expression and somatic mutation data of TCGA-LIHC were downloaded from https://xenabrowser.net . The clinical and mRNA expression data of the GSE76427 and ICGC-LIRI cohorts were downloaded from https://www.ncbi.nlm.nih.gov/geo/ and https://dcc.icgc.org/projects/, respectively.

The RNA data (FPKM or count format) were transformed into TPM format. The batch effects among the TCGA-LIHC, GSE76427 and ICGC-LIRI cohorts were eliminated via the SVA” package (Yang et al., 2020). The CNV diagram of 16 cuproptosis-related genes was generated by the “Rcircos” package.

### Identification of HCC cuproptosis subtypes (Cu-clusters) based on NMF unsupervised clustering analysis

The 16 cuproptosis-related genes were obtained from published literatures, including 13 genes with positive relationships (FDX1, LIPT1, LIAS, DLD, DBT, GCSH, DLST, DLAT, PDHA1, PDHB, SLC31A1, ATP7A and ATP7B) and 3 genes with negative relationships (CDKN2A, MTF1 and GLS). Based on the expression of these 16 cuproptosis-related genes, we performed an unsupervised clustering analysis (NMF) and identified three different cuproptosis-related subtypes. The clustering analysis was performed with the “consensus-cluster plus” package and iterated 1,000 times (Wilkerson and Hayes, 2010).

### Pathway enrichment analysis of hallmark and GO gene sets

The hallmark gene sets were downloaded from the MSigDB (http://www.gsea-msigdb.org). Single-sample enrichment scores for the hallmark gene set were estimated with the “GSVA” package (Hanzelmann et al., 2013). The package “clusterProfiler” was applied to annotate the GO functions of DEGs.

### Estimation of immune cell infiltration with the CIBERSORT and MCPcounter algorithms

The “MCPcounter” package and CIBERSORT algorithm were employed to estimate the fractions of tumor-infiltrating immune cell subsets based on mRNA transcriptome profiles (Becht et al., 2016). The CIBERSORT algorithm (https://cibersort.stanford.edu/) can be used to evaluate the abundance of 22 immune cells, and the MCPcounter algorithm can be used to quantify the fractions of 8 tumor-infiltrating immune cell types, as well as endothelial cells and fibroblasts.

### Identification of cuproptosis subtype-related DEGs

The “limma” package was applied to screen DEGs. A total of 140 DEGs were identified with significance criteria (adjusted *p* value < 0.001, |logFC>1|). The “heatmap” package was used to present the expression landscape of DEGs among three different cuproptosis subtypes.

### Construction of a prognostic signature based on the cuproptosis-related DEGs

The “caret” package was used to divide the patients **(**from the TCGA-LIHC and GSE76427 cohorts) into training (70%) and testing (30%) cohorts. The independent cohort ICGC-LIRI was used for validation. The “survival” and “glmnet” packages were used for the univariate Cox and LASSO Cox analyses to identify the cuproptosis-related and prognostically significant hub genes. The cuproptosis-related prognostic signature (termed Cu-PS) was constructed by multivariate Cox regression. The formula is as follows: Cu-PS = $\overset{n}{\underset{i}{\sum }}coef_{i}*mRNA_{i}$ . The selection of the optimal cutoff value was based on the “surv_cutpoint” function in the “survival” package. The “survivalROC” and “survminer” packages were used to generate the receiver operating characteristic (ROC) and Kaplan–Meier (K−M) curves, respectively.

### Quantitative real-time PCR (qRT–PCR) in cell lines

To validate the expression levels of the 5 hub genes in normal and HCC cell lines, we cultured two HCC cell lines (Huh7 and HLE) and one human hepatocellular cell line (MIHA). Total RNA was extracted from the above 3 cell lines, and cDNA was synthesized with a reverse transcription kit (TaKaRa, Japan). The qRT–PCR was performed using SYBR Green Mix (TaKaRa, Japan) and a C1000 system (Bio–Rad, Hercules, CA). The primer sequences of the 5 hub genes are listed in Table 1. The RNA quality was assessed, and RNA levels were normalized based on human GAPDH.

**TABLE 1:** The primer sequences of the 5 hub genes.

Genes	Primer sequence (5′-3′)
C7 (human)-F	AAT​GGC​TGT​ACC​AAG​ACT​CAG​A
C7 (human)-R	GCT​GAT​GCA​CTG​ACC​TGA​AAA
MAGEA6 (human)-F	AGG​GGA​GGG​AAG​ACA​GTA​TCT
MAGEA6 (human)-R	AAA​GCC​CAC​TCA​TGC​AGG​AG
HK2(human)-F	GAG​CCA​CCA​CTC​ACC​CTA​CT
HK2(human)-R	CCA​GGC​ATT​CGG​CAA​TGT​G
CYP26B1(human)-F	GGC​AAC​GTG​TTC​AAG​ACG​C
CYP26B1(human)-R	TGC​TCG​CCC​ATG​AGG​ATC​T
EPO (human)-F	GGA​GGC​CGA​GAA​TAT​CAC​GAC
EPO (human)-R	CCC​TGC​CAG​ACT​TCT​ACG​G

### Ability of the Cu-PS to predict immunotherapy response

The Tumor Immune Dysfunction and Exclusion (TIDE) algorithm was employed to quantify immunosuppressive and dysfunctional factors in the tumor immune microenvironment (TIME) and to estimate the ability of cancer cells to escape antitumor immunity (Jiang et al., 2018). In addition, the IMvigor210 cohort (Mariathasan et al., 2018) (348 urothelial carcinoma patients) and Liu et al. cohorts (Liu et al., 2019) (121 melanoma patients) were employed to validate the relationship between the Cu-PS and immunotherapy response. The mRNA data of the two immunotherapy cohorts were transformed into TPM values before further analysis.

### Analysis of the correlation of the Cu-PS with drug sensitivity

The “pRRophetic” package (Geeleher et al., 2014) was used to calculate the IC50 values of drugs in the Genomics of Drug Sensitivity in Cancer (GDSC) database. We analyzed the correlation between IC50 values and the Cu-PS with Spearman correlation analysis to explore the association of the Cu-PS with drug sensitivity. |Cor| > 0.2 and adjusted *p* < 0.05 were used as cutoffs for identifying significant correlations.

### Statistical analyses

All statistical analyses were performed with the software of R-4.0.2. The Wilcoxon rank-sum test and Kruskal–Wallis tests were applied to identify differences between two and among three groups, respectively (Hazra and Gogtay, 2016). K−M analysis and the log-rank test were utilized to analyze differences between distinct Cu-clusters, Cg-clusters and Cu-PS subgroups. The mutation waterfall plot was generated with the “maftools” package (Mayakonda et al., 2018). The CNV circle graph of 16 cuproptosis-related genes in human chromosomes was generated with the “RCircos” package (Zhang et al., 2013). All tests were bilateral, *p* < 0.05 was considered significant, and the false discovery rate (FDR) was calculated for multiple hypothesis testing (Ferreira, 2007).

## Results

### Multiomics landscape of 16 cuproptosis-related genes and identification of cuproptosis subtypes in HCC

**FIGURE 1:**
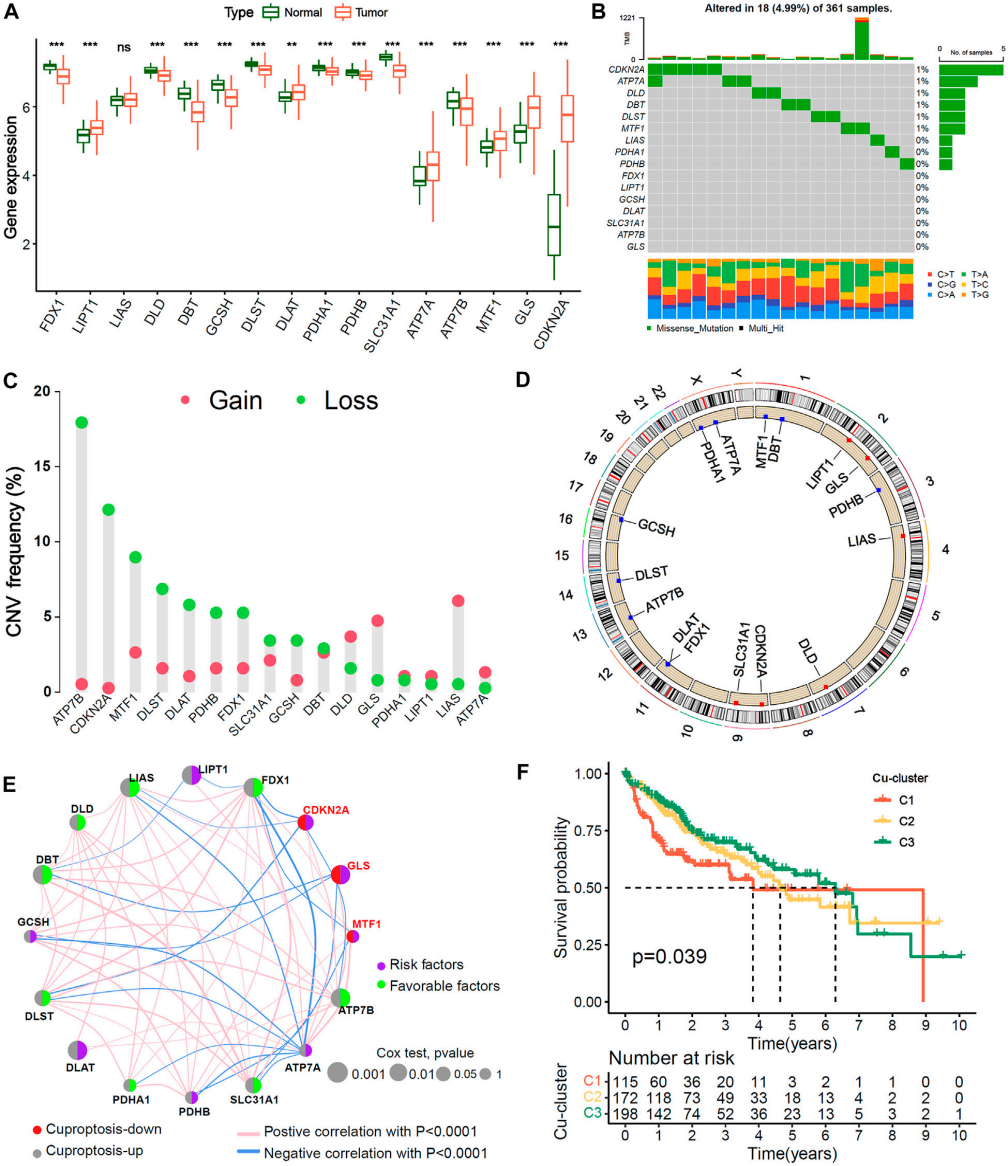
Genetic variation landscape and Unsupervised clustering of 16 Cu-RGs in HCC. **(A)** Comparison of 16 Cuproptosis-related genes (Cu-RGs) between normal and HCC tissues in mRNA expression. **(B)** The mutation frequency and classification of 16 Cu-RGs in TCGA-LIHC cohort. **(C)** The CNV variation frequency of 16 Cu-RGs in TCGA-LIHC cohort. **(D)** The location of CNV alteration of 16 Cu-RGs on the chromosomes in TCGA-LIHC. **(E)** Co-expression and prognosis relationship of 16 Cu-RGs in TCGA-LIHC. The regulation of Cu-RGs to cuproptosis were depicted by circles (lift) in two colors: red, cuproptosis-down; gray, cuproptosis-up. The lines connecting 16 Cu-RGs represented their interaction with each other. The size of each circle represented the prognosis effect of each regulator and scaled by *p*-value. The color on the right half of the circle represents the effect of Cu-RGs on prognosis: green, protective factors; purple, risk factors. **(F)** Kaplan-Meier curves of overall survival (OS) for 485 HCC patients in TCGA-GEO cohort with different Cuproptosis-clusters (termed as C1, C2 and C3). The numbers of patients in C1, C2, and C3 are 115, 172, and 198, respectively.

The study analysis flowchart was shown in Supplementary Figure S1. First, we analyzed the differences in the mRNA expression of 16 cuproptosis genes between HCC and normal liver tissues. The results showed that in HCC patients, 9 of 13 genes positively associated with cuproptosis were expressed at lower levels, 3 of 13 cuproptosis positive genes (LIPT1, DLAT and ATP7A) and the 3 cuproptosis negative genes (GLS, MTF1 and CDKN2A) were upregulated, indicating that the level of cuproptosis was lower in HCC than in normal liver tissues (Figure 1A). The mutation analysis showed that 18 of 361 (4.99%) HCC patients had mutations in cuproptosis-related genes (Figure 1B). The copy number alteration (CNA) frequency analysis showed that most of the cuproptosis genes had deletions affecting copy number. ATP7B had the highest frequency of deletion, while LIAS had the highest frequency of amplification (Figure 1C). The chromosomal locations of the 16 cuproptosis genes CNA are shown in Figure 1D.

TCGA-LIHC and GSE76427 samples with available survival information were employed to generate the comprehensive crosstalk network of the 16 cuproptosis genes (Figure 1E). We found that the three genes negatively associated with cuproptosis (CDKN2A, MTF1 and GLS) were risk factors for HCC overall survival (OS) (HR < 1, univariate Cox test), and most of the genes positively associated with cuproptosis were favorable factors (HR < 1, univariate Cox test), which indicated that HCC patients may benefit from cuproptosis. The NMF algorithm was used to classify 485 HCC samples into three distinct cuproptosis subgroups, termed Cu-cluster 1, Cu-cluster 2 and Cu-cluster 3, based on the expression of the 16 cuproptosis genes. The process of cluster analysis (rank = 2:7) is shown in Supplementary Figure S2. K−M survival analysis showed that the patients in Cu-cluster 3 had the best OS benefit, followed by those in Cu-cluster 2 and Cu-cluster 1 (*p* = 0.039, Figure 1F). We performed a multi-genetic analysis and identified three distinct cuproptosis-subgroups associated with different OS prognoses.

### Analysis of enriched pathways and immune cell infiltration among the three Cu-clusters

**FIGURE 2:**
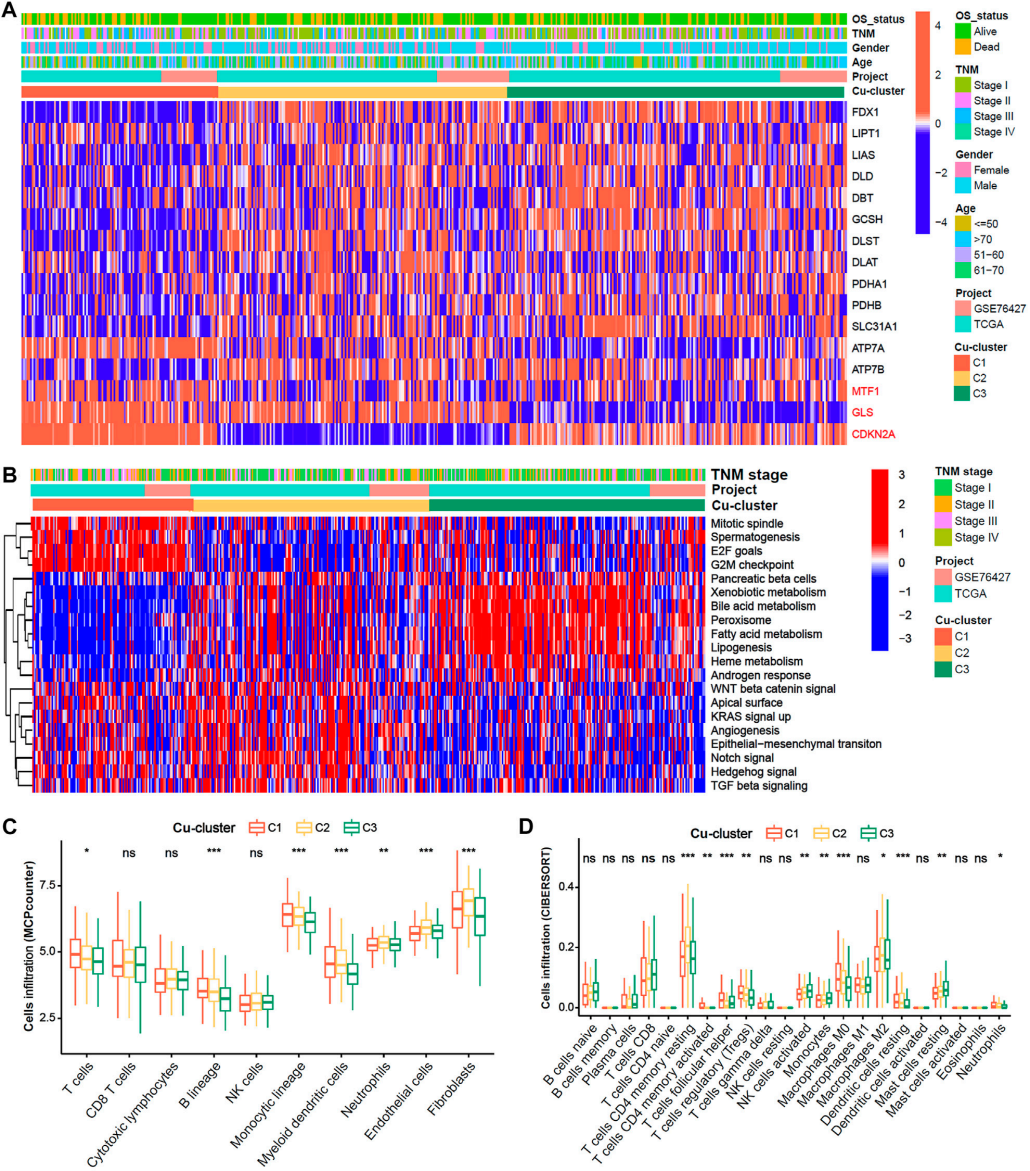
Biological characteristics and immune cell infiltration characteristics in distinct cuproptosis-clusters. **(A)** Expression heatmap of 16 Cu-RGs in the TCGA-GEO cohort. The patient annotations of Cuproptosis-cluster, OS-status, gender, TNM stage, age and cohorts were presented at the top of heatmap. **(B)** GSVA score heatmap of Hallmark pathways among three Cuproptosis-clusters. **(C,D)** Comparison of immune cell infiltration among three Cuproptosis-clusters, which was estimated by MCPcounter **(C)** and CIBERSORT **(D)** algorithm, respectively.

We explore the cuproptosis level based on the expression of the 16 cuproptosis-related genes among the three Cu-clusters. The patients in Cu-cluster 1 had the lowest cuproptosis level, as indicated by lower expression of genes positively associated with cuproptosis and overexpression of genes negatively associated with cuproptosis. The patients in Cu-cluster 2 had a moderate cuproptosis level, and those in Cu-cluster 3 had the highest cuproptosis level (Figure 2A). To discover the underlying biological mechanisms behind the differences in survival between the three Cu-clusters, we performed gene set enrichment analysis (GSEA) and immune cell infiltration analysis. The GSEA results showed that Cu-cluster 1 was enriched in mTORC1 signaling, E2F targets and G2/M checkpoint pathways. Cu-cluster 3 was associated with metabolic pathway terms, such as bile acid metabolism, peroxisome, lipogenesis and fatty acid metabolism. Cu-cluster 2 was enriched in the terms Notch signaling, angiogenesis, epithelial-mesenchymal transition and TGF-β signaling (Figure 2B). We further analyzed immune cell infiltration with MCPcounter (Figure 2C) and the CIBERSORT algorithm (Figure 2D). The HCC patients in Cu-cluster 1 had the highest levels of inhibitory immune cells (myeloid dendritic cells (DCs), regulatory T cells (Tregs) and M0 macrophages), while those in Cu-cluster 2 had the highest levels of stromal cell subsets (endothelial cells and fibroblasts); those in Cu-cluster 3 had lower immune cell infiltration. Furthermore, the patients in cluster-C3 and normal samples were less infiltrated in T cell, B cell as well as endothelial cells and fibroblast (Supplementary Figure S3). These results indicated that cuproptosis subtype is associated with tumor microenvironment factors.

### Identification of cuproptosis-related and prognosis-related hub genes in HCC

**FIGURE 3:**
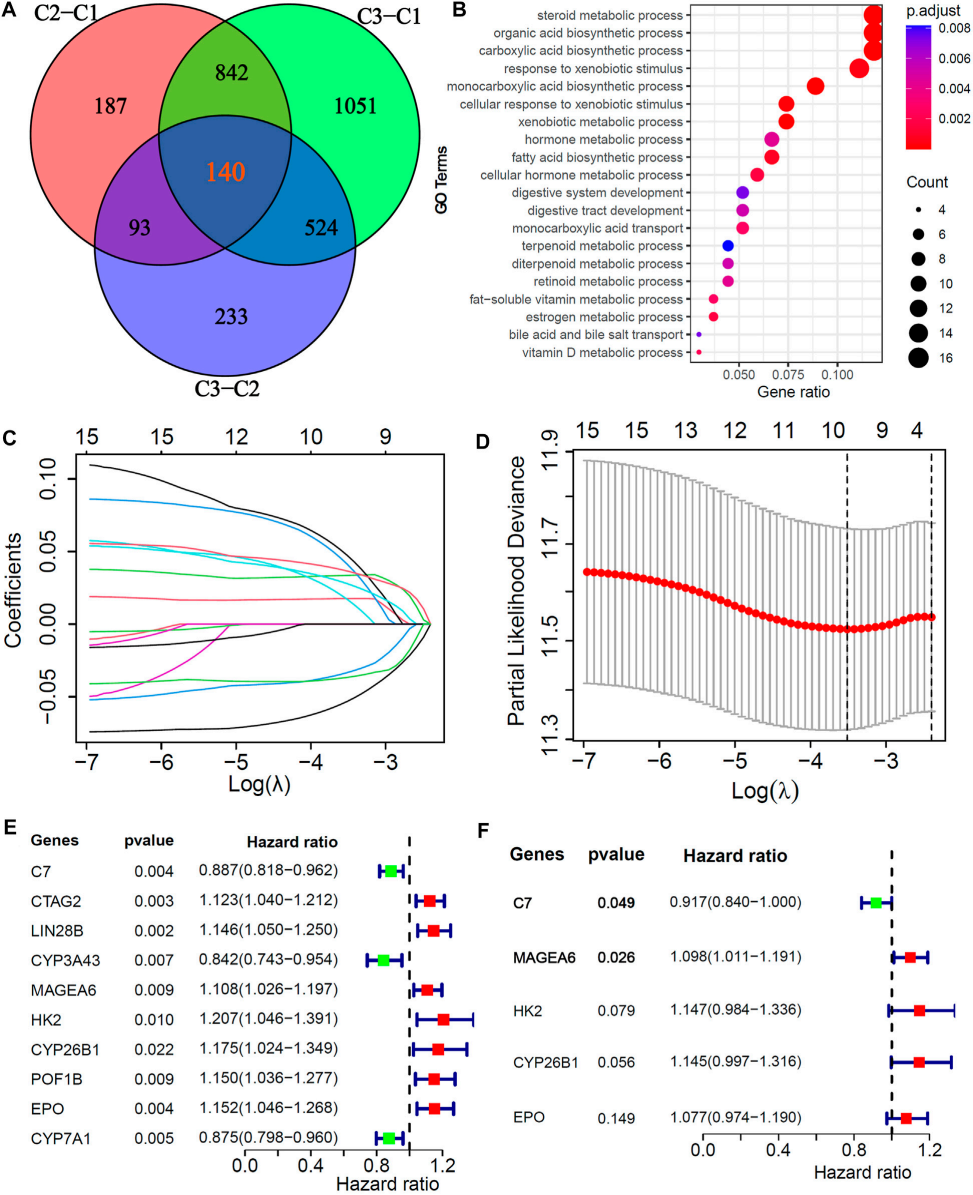
Screening of Cu-DEGs and construction of cuproptosis-related prognostic signatures (Cu-PS) **(A)** The Venn diagram illustrated the 140 Cu-DEGs among three cuproptosis-clusters. **(B)** GO enrichment analysis revealed the biological characteristics of the 140 DEGs. **(C)** 10 Cu-DEGs screened by LASSO regression. **(D)** 10-fold cross-validation plot of LASSO regression. **(E)** Univariate Cox forest-plot of the 10 selected Cu-DEGs. **(F)** Forest-plot of 5 hub Cu-DEGs in Cu-PS identified by multivariate Cox regression analysis.

To explore potential biological behaviors, we identified 140 DEGs among the three subtypes (|logFC|>0.5, adjusted *p* < 0.01, Figure 3A) and further performed GO enrichment analysis. The results showed that the DEGs were mainly enriched in metabolic processes, such as steroid metabolic processes, xenobiotic metabolic processes, fatty acid biosynthetic processes, cellular hormone metabolic processes, estrogen metabolic processes, vitamin D metabolic processes and bile acid and bile salt transport (Figure 3B). In the training cohort, univariate, LASSO and multivariate Cox analyses were applied to identify the cuproptosis-related and prognosis-related hub genes. A total of 10 prognostic DEGs were selected through LASSO Cox analysis (Figures 3C,D), and 5 prognostic hub DEGs were identified *via* multivariate Cox analysis. The hazard ratios and *p* values of these selected genes are shown in the forest plot of the univariate Cox analysis results (Figures 3E,F). One hub DEG (C7) was the favorable factor for HCC prognosis, while the other hub DEGs (MAGEA6, HK2, CYP26B1 and EPO) were risk factors.

### Construction and validation of the cuproptosis-related prognostic signature in HCC

**FIGURE 4:**
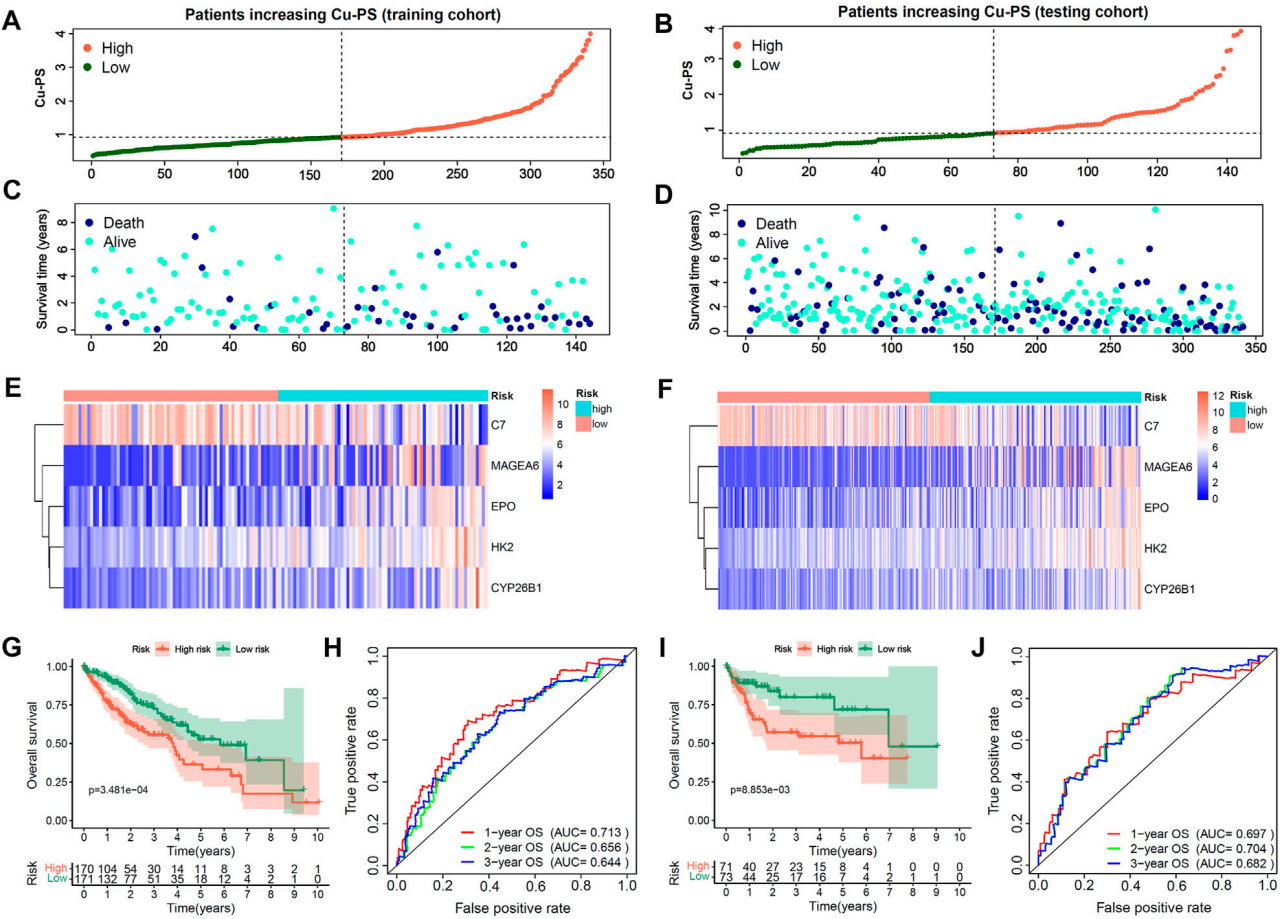
Construction and validation of Cu-PS in training and testing cohorts. **(A,B)** The range of Cu-PS in the training and testing cohorts, and patients were cutoff into high- and low-two subgroups by the and median value of Cu-PS. **(C,D)** The distribution plots showed the survival status of patients with increasing Cu-PS in the training and testing cohorts. **(E,F)** The heatmaps showed the expression of 5 hub Cu-DEGs between two Cu-PS subgroups in the training and testing cohorts. **(G,H)** The Kaplan-Meier curves of overall survival (OS) between the two subgroups in the training and testing cohorts. **(I,J)** The ROC curves of the Cu-PS in predicting 1-, 2-, and 3-year OS in the training and testing cohorts.

With the training cohort, we further constructed a cuproptosis-related prognostic signature termed the Cu-PS *via* multivariate Cox analysis. The formula used to calculate the Cu-PS is described in the Methods section. The Cu-PS the training cohort was calculated with the same formula. The patients with an increased Cu-PS had a high fraction of death and shortened survival time in both the training cohort and testing cohort (Figures 4A–D). As can be seen in the heatmaps, the risk genes (MAGEA6, HK2, CYP26B1 and EPO) were upregulated with increasing Cu-PS, while the protective gene C7 was downregulated (Figures 4E,F). HCC samples were divided into high- and low-risk groups based on the median Cu-PS. The HCC patients in the training cohort with low Cu-PS had longer OS, and a similar result was found in the testing cohort (Figures 4G,I; *p* < 0.001). The AUCs of Cu-PS for predicting 1-, 2-, and 3-year survival were 0.697, 0.704, and 0.682, respectively, in the training cohort and 0.713, 0.656, and 0.644, respectively, in the testing cohort (Figures 4H,J).

**FIGURE 5:**
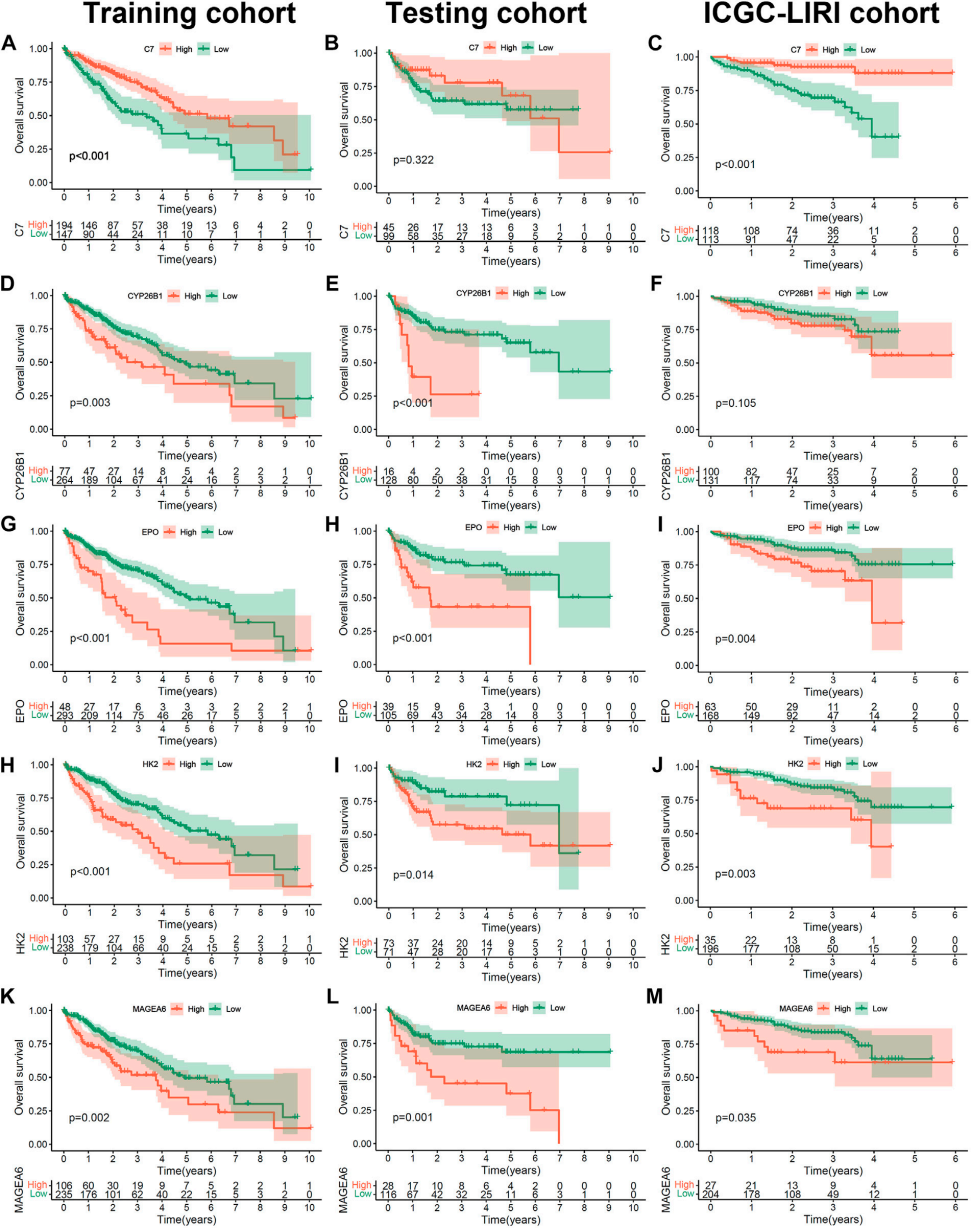
The prognosis value of 5 hub Cu-DEGs in three HCC cohorts. **(A–M)** The Kaplan-Meier curves of C7 in training **(A)**, testing **(B)** and ICGC **(C)** cohorts, as well as CYP26B1**(D–F)**, EPO **(G–I)**, HK2 **(H–J)** and MAGEA6 **(K–M)**.

To further confirm the prognostic value of the 5 hub genes (C7, MAGEA6, HK2, CYP26B1 and EPO) in HCC, we performed K-M survival analysis in the training cohort, testing cohort and independent ICGC-LIRI cohort (Figures 5A–M). The optimal cutoff value was identified by the “cutpoint” function in the “survival” package. Consistent with the previous results (Figure 3F), the patients with high expression of MAGEA6, HK2, CYP26B1 and EPO had a worse prognosis in the training, testing and ICGC-LIRI cohorts (Figures 5D,E,G–M and G-M, all *p* < 0.05; Figure 5F, *p* = 0.105). Meanwhile, patients with high C7 expression lead a prognostic benefit in the training cohort and ICGC-LIRI cohort (Figures 5A,C; both *p* < 0.001), and the same trend was found in the testing cohort (Figure 5B, *p* = 0.322).

We further validated the 5 hub genes in tissues (TCGA-LIHC dataset) and cultured normal (MIHA) and HCC cell lines (Huh7 and HLE). The results from the TCGA-LIHC dataset showed that C7, CYP26B1 and EPO were significantly downregulated in HCC, while MAGEA6 was significantly upregulated (Supplementary Figure S4A). Similar results were found in the cell lines, but EPO and CYP26B1 did not show the same differences (Supplementary Figure S4B). The expression of EPO in HCC cell lines tended to be higher (without a significant difference) than that in the normal cell line. The expression of CYP26B1 in MIHA and Huh7 cell lines was significantly higher than that in the HLE cell line.

### Analysis of the correlation of the Cu-PS with clinical characteristics and TMB

**FIGURE 6:**
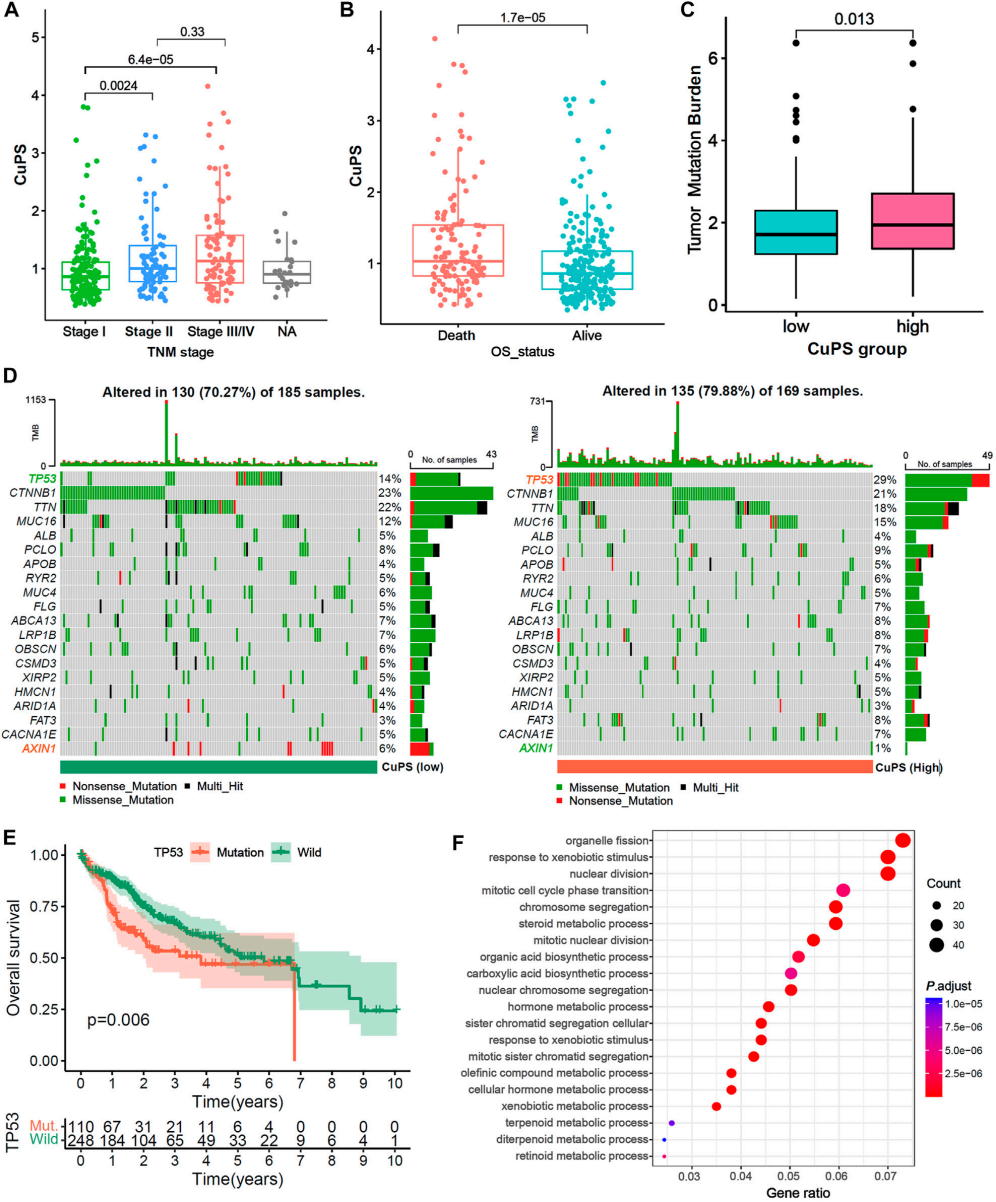
Correlation between the Cu-PS and clinical features and the landscape of somatic mutation. **(A,B)** Comparison of Cu-PS among different TNM stages **(A)** and survival outcomes **(B)**. Differences in risk scores between different survival outcome. **(C)** Comparison of TMB value between high- and low- Cu-PS subgroups. **(D)** The waterfall plots showed the somatic mutation spectrum of the high- and low-risk groups. **(E)** Kaplan-Meier curves between TP53 mutation (110 patients) and wild (248 patients) groups in TCGA-LIHC. **(F)** GO enrichment analysis of the DEGs between high- and low- Cu-PS subgroups.

Patients with TNM stage Ⅱ and Ⅲ/Ⅳ disease had a higher Cu-PS than those with stage Ⅰ disease (Figure 6A, both *p* < 0.01). In addition, patients who died had a higher Cu-PS than those who survived (Figure 6B, *p* < 0.001). Moreover, the TMB value in the high-Cu-PS group was significantly higher than that in the low-Cu-PS group (Figure 6C, *p* = 0.013). Waterfall plots of the top 20 frequently mutated genes are shown in Figure 6D. The patients with high Cu-PS had a significantly higher mutation frequency of TP53 but a lower frequency of AXIN1 mutation. As expected, the patients with TP53 mutation had a significant OS benefit compared with TP53 wild-type patients (Figure 6E, *p* = 0.006). We further performed GO enrichment analysis of the DEGs between the high- and low-Cu-PS groups. The results showed that the DEGs were mainly enriched in the cell cycle and metabolic processes, such as nuclear division, mitotic cell cycle phase transition, chromosome segregation, organic acid biosynthetic processes, carboxylic acid biosynthetic processes, and hormone metabolic processes (Figure 6F). These findings confirmed the prognosis values and revealed the underlying mechanism of Cu-PS in HCC.

### Ability of the Cu-PS to predict drug sensitivity and immunotherapy efficacy

**FIGURE 7:**
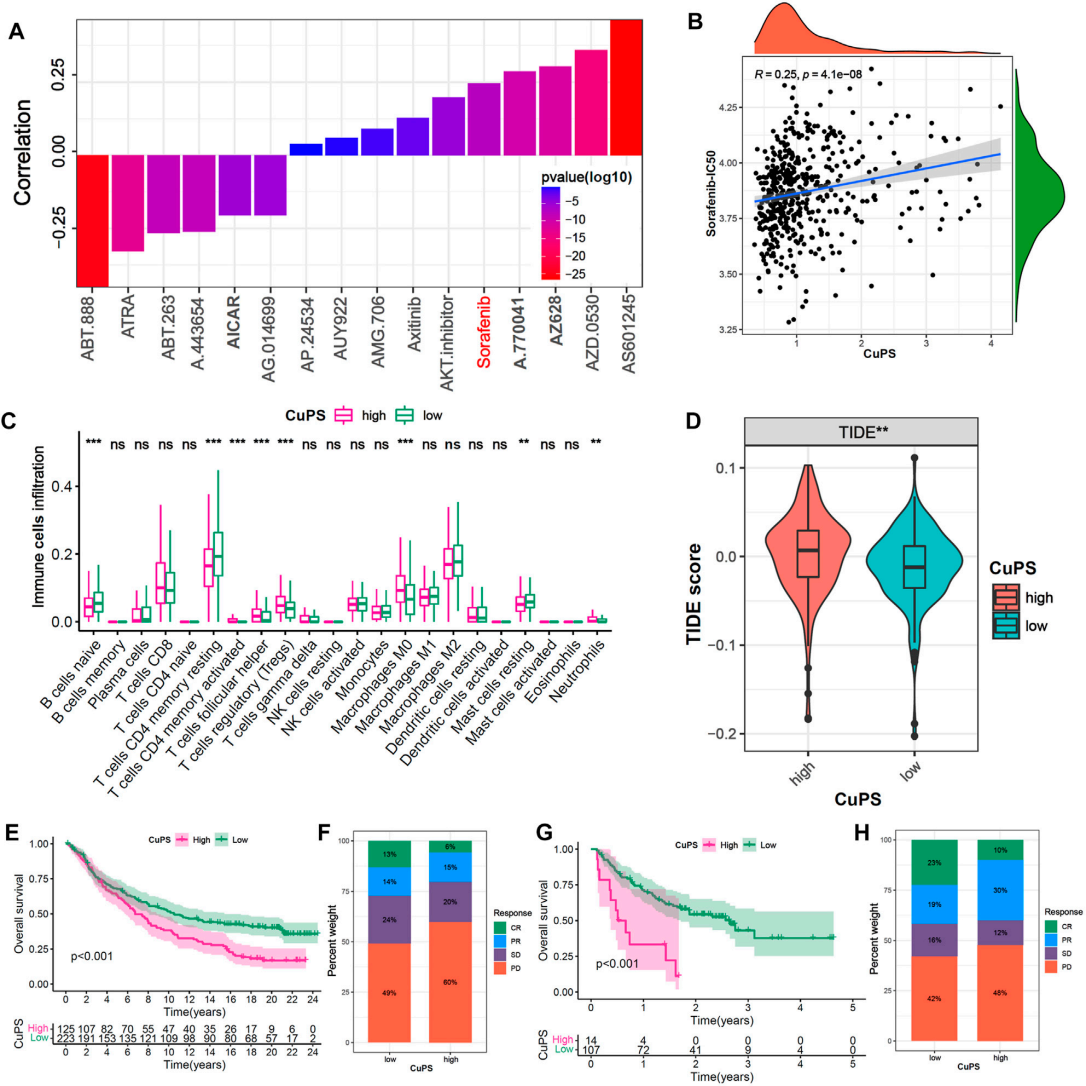
Efficacy prediction of chemotherapy drugs and immunotherapy. **(A)** The correlation of Cu-PS with IC50 of drugs in GDSC database. **(B)** The correlation scatter plot of Cu-PS and Sorafenib-IC50. **(C)** The proportion of immune cells in TME between the high- and low- Cu-PS subgroups. **(D)** Comparison of TIDE score between the two Cu-PS subgroups. **(E)** Kaplan-Meier curves for high- and low- Cu-PS subgroups in the anti-PD-L1 immunotherapy cohort (IMvigor210). **(F)** The proportion of immune response (CR, PR, SD and PD) between two Cu-PS subgroups in IMvigor210 cohort. **(G)** Kaplan-Meier curves for high- and low- Cu-PS subgroups in the anti-PD1 immunotherapy cohort (GSE78220 cohort) **(H)** The proportion of immune response between two Cu-PS subgroups in GSE78220 cohort.

To further investigate the potential application value of the Cu-PS in HCC treatment, we explored the correlations with drug sensitivity and immunotherapy efficacy based on the GDSC database and two immunotherapy cohorts (the GSE78220 and IMvigor210 cohorts). We identified 16 drugs in the GDSC database that were significantly associated with the Cu-PS by Spearman correlation analysis (Figure 7A, *p* < 0.05). Among them, 10 drugs showed a correlation between drug sensitivity and the Cu-PS, including sorafenib, the Src inhibitor A.770,041, the RAF inhibitor AZ628, the Src/Abl dual-kinase inhibitor AZD.0530, and the JNK inhibitor AS601245 (all cor>0.2, all *p* < 0.05). Six drugs showed a correlation between the Cu-PS and drug resistance, including the PARP inhibitor ABT-888, all-trans retinoic acid (ATRA), the Bcl-2 inhibitor ABT.263, the Akt inhibitor A.443654 and the AMPK activator (all cor<0.2, all *p* < 0.05). Sorafenib is a first-line treatment for advanced HCC. As expected, the IC50 of sorafenib was significantly positively associated with the Cu-PS (Figure 7B, cor = 0.25, *p* < 0.001), which was in line with the sensitivity of HCC to sorafenib. These results imply that the Cu-PS is correlated with drug sensitivity and might be a potential biomarker for guiding drug treatment selection.

Immunotherapy has made major breakthroughs in the treatment of liver cancer. The immune cell infiltration analysis showed that the patients with a high Cu-PS had a suppressive immune microenvironment, poor prognosis, and significantly enriched levels of Tregs, M0 macrophages, and neutrophils but decreased levels of B cells, CD4^+^ T cells and mast cells (Figure 7C). In addition, the patients in low-Cu-PS group had a high fraction of memory CD8 T cell, while the effector CD8 T cell was not significant (Supplementary Figure S5). The TIDE scores in the high-Cu-PS group were also significantly higher than those in the low-Cu-PS group, which was associated with resistance to immunotherapy (Figure 7D). We further confirmed the predictive ability of the Cu-PS in two immunotherapy cohorts (the IMvigor210 and Liu cohorts). The results showed that the patients in the Cu-PS-low group in the IMvigor210 cohort had better OS (Figure 7E, *p* < 0.001). The fractions of patients who achieved complete response (CR) and partial response (PR) in the Cu-PS-low group were higher than those in the Cu-PS-high group (Figure 7F). Similar results were found in the Liu cohorts (Figures 7G,H). In summary, these findings suggest that application of the Cu-PS might improve drug selection and immunotherapy response prediction in HCC.

## Discussion

Cuproptosis is a newly discovered PCD process, and copper metabolism plays an important role in tumor development, invasion and metastasis. In this study, we systematically performed multiomics bioinformatics analyses to explore the association of cuproptosis-related genes with genomic and TIME characteristics, prognosis and immunotherapy response in HCC.

In this study, we assessed the relevance of cuproptosis and the immune microenvironment in HCC. We performed multiomics analysis of the 16 cuproptosis-related genes and found that the level of cuproptosis was significantly higher in normal liver tissues than in HCC tissues, which indicated that cuproptosis may suppress tumorigenesis to a certain extent. We further identified three distinct cuproptosis-related subgroups (Cu-clusters) associated with OS and with different enriched tumor hallmark pathways. Cu-cluster 1 was enriched in mTORC1 signaling, E2F targets and G2/M checkpoint pathways. Cu-cluster 3 was enriched in metabolism pathways, while Cu-cluster 2 was enriched in angiogenesis and epithelial-mesenchymal transition pathways. The HCC patients in Cu-cluster 1 had the highest levels of inhibitory immune cells (myeloid DCs, Tregs and M0-macrophages), while Cu-cluster 2 had the highest levels of stromal cell subsets (endothelial cells and fibroblasts), and Cu-cluster 3 had lower immune cell infiltration.

We further constructed a 5-gene (C7, MAGEA6, HK2, CYP26B1 and EPO) prognostic signature termed the Cu-PS *via* univariate Cox, LASSO and multivariate Cox regression analyses. Complement component 7 (C7) is an essential component of the complement system (CS) and is involved in membrane attack complex (MAC) formation. Moreover, the C7 peptide can inhibit Akt and Erk1/2 and further suppress HCC cell migration and invasion induced by HGF (Zhao et al., 2019).

Melanoma-associated antigen family A (MAGEA) antigens are expressed in a variety of malignancies. MAGEA6 can promote pancreatic (Tsang et al., 2020), lung cancer (Pineda et al., 2015) and colorectal cancer (Wu et al., 2018) carcinogenesis by inhibiting autophagy. In addition, MAGEA6 regulates stemness and self-renewal in HCC by activating the AMPK signaling pathway and is associated with poor prognosis (Guo et al., 2019).

Pericyte-hexokinase 2 (HK2) is the key rate-limiting enzyme of the glycolytic pathway, which is associated with pathological stage and prognosis (DeWaal et al., 2018). In the mouse model of HCC, HK2 silencing could synergistically enhance the sensitivity of HCC cells to sorafenib (Yu et al., 2022). CYP26 enzymes are the major enzymes responsible for the clearance of retinoic acid (RA), which is an important endogenous signaling molecule that regulates the cell cycle and maintains epithelial cells. CYP26 inhibitors can enhance endogenous RA activity in a cell-type-specific manner and might be new, attractive targets in cancer and skin disease treatment (Tsvetkov et al., 2022). Erythropoietin (EPO) is primarily synthesized in the kidney and can promote erythrocyte production. Under hypoxic conditions, EPO is upregulated to promote the production of red blood cells and enhance the oxygen-carrying capacity (Liu et al., 2020).

Although we conducted a comprehensive and systematic analysis, the identification of additional new cuproptosis-related genes will enrich our research. Due to the lack of an HCC immunotherapy cohort, we assessed the ability of a prognostic signature (Cu-PS) to predict the efficacy of immunotherapy in two cohorts of patients with different cancers treated with immunotherapy. In conclusion, we performed a comprehensive analysis of 16 cuproptosis-related genes in 716 HCC samples and identified a novel HCC prognostic signature (Cu-PS), providing a new strategy for predicting HCC prognosis and immunotherapy efficacy.

## Conclusion

Compared with already known biomarkers such as AFP, GPC3 and DCP, our study constructed a novel cuproptosis-related prognostic signature (Cu-PS) that might be a useful biomarker for predicting immunotherapy response and enhancing diagnosis and treatment of HCC, which indicates that cuproptosis is associated with the TIME and HCC prognosis.

## Data Availability

The original contributions presented in the study are included in the article/Supplementary Material, further inquiries can be directed to the corresponding author.
