# Supplementary material for: A novel cuproptosis-related prognostic signature and potential value in HCC immunotherapy
Source: Front Mol Biosci. 2022 Sep 29;9:1001788. doi: 10.3389/fmolb.2022.1001788 (PMC9556951; doi:10.3389/fmolb.2022.1001788)
Supplement: Supplementary file 1 [file DataSheet1.docx]

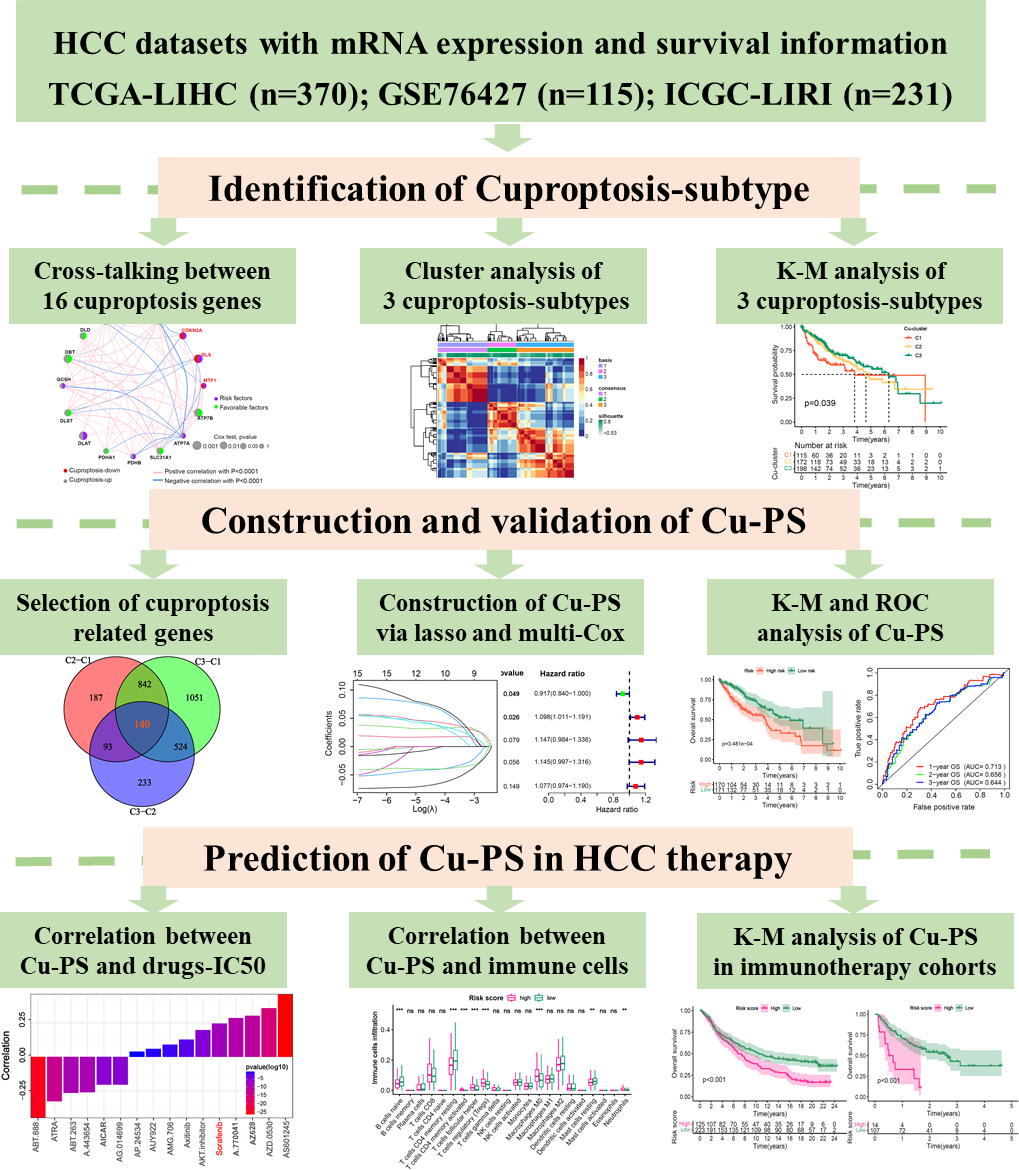


**Figure S1. The study analysis flowchart.**

A total of 716 HCC samples with complete survival and mRNA expression information from three HCC cohorts (TCGA-LIHC cohort, n=370; GSE76427 cohort, n=115; ICGC-LIRI cohort, n=231) were included in this study. We first analyzed the mutation landscape of cuproptosis-related genes in HCC. Based on the expression of 16 cuproptosis-related genes, HCC samples were divided into three subgroups by nonnegative matrix factorization (NMF) clustering. Differential pathway enrichment and immune cell infiltration analyses of these three subgroups were conducted to explore the biological mechanisms underlying the three subtypes. The cuproptosis-related prognostic signature (Cu-PS) was established based on the five identified prognostic core genes. In addition, the prognostic values of Cu-PS and five core genes were further validated in testing and ICGC-LIRI cohorts. Furthermore, the Cu-PS was significantly associated with drug sensitivity, immune microenvironment and immunotherapy response.


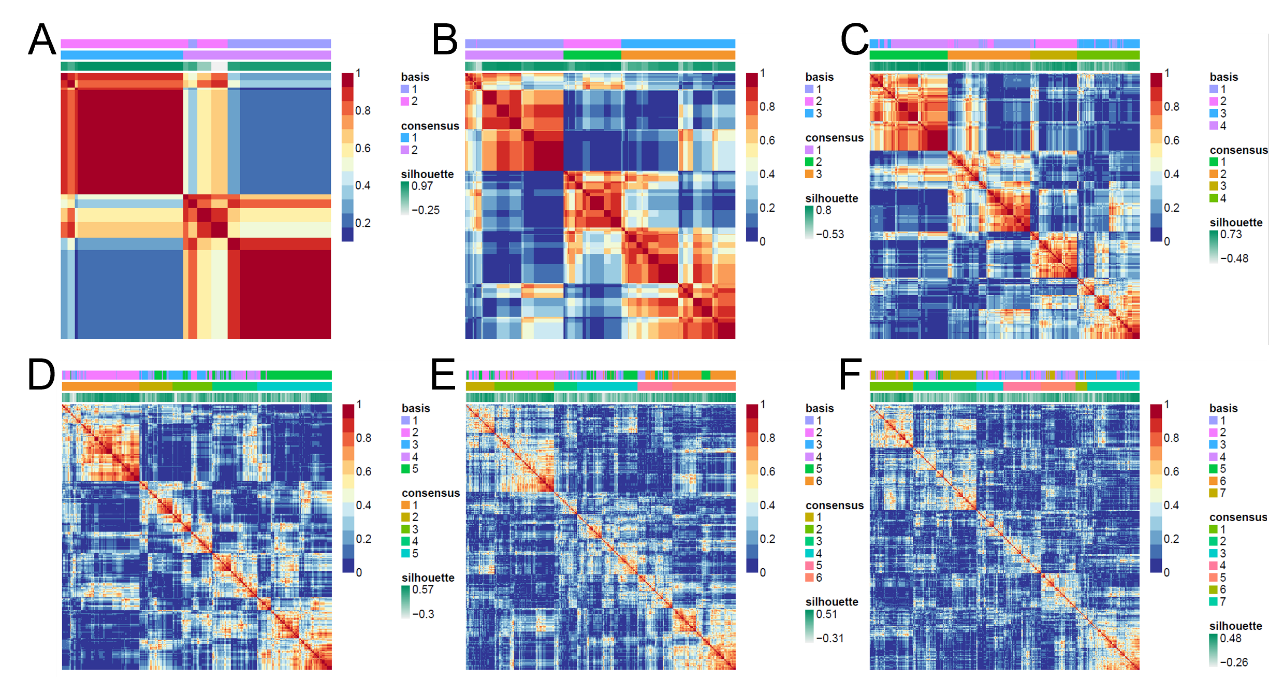


**Figure S2. Heatmap of NMF clustering for 16 CRG in TCGA-GEO cohort.**

(A-F) Heatmap of NMF clustering for 16 Cu-RGs in TCGA-GEO cohort with cluster numbers from 2 to 7.


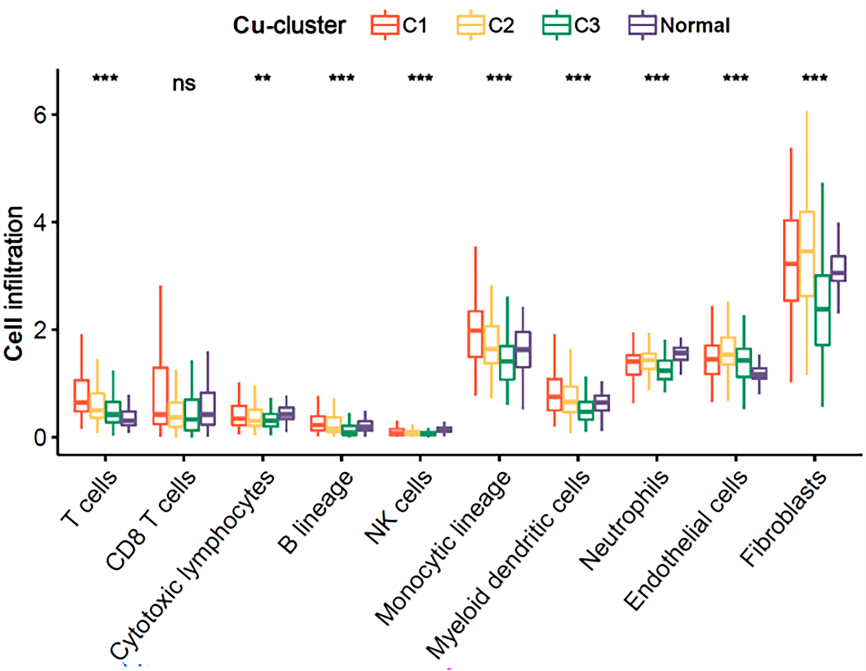


**Figure S3.** **Comparison of cell infiltration among three Cuproptosis-clusters and normal samples.** The bar in red, yellow, green and grey were presented Cu-cluster C1, C2, C3 and normal samples.


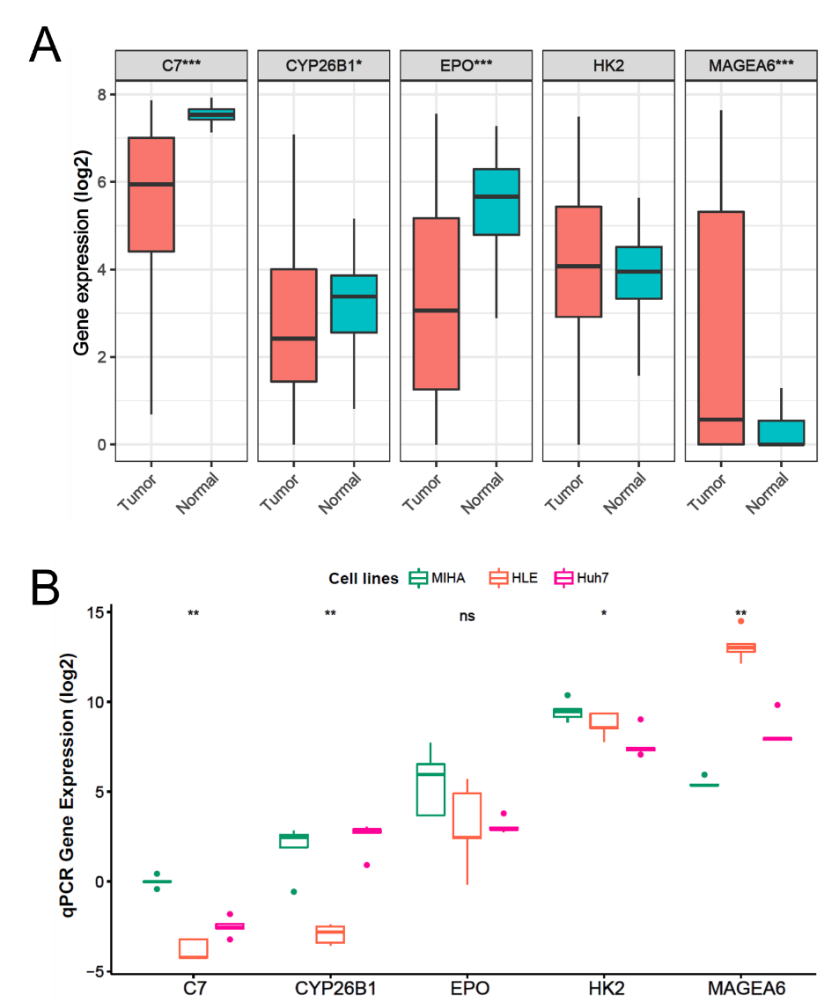


**Figure S4. The expression level of 5 hub Cu-DEGs in TCGA(A) and cell lines(B).**

(A-B) Expression comparison of 5 hub Cu-DEGs between normal and HCC in TCGA(A) and cell lines (B).


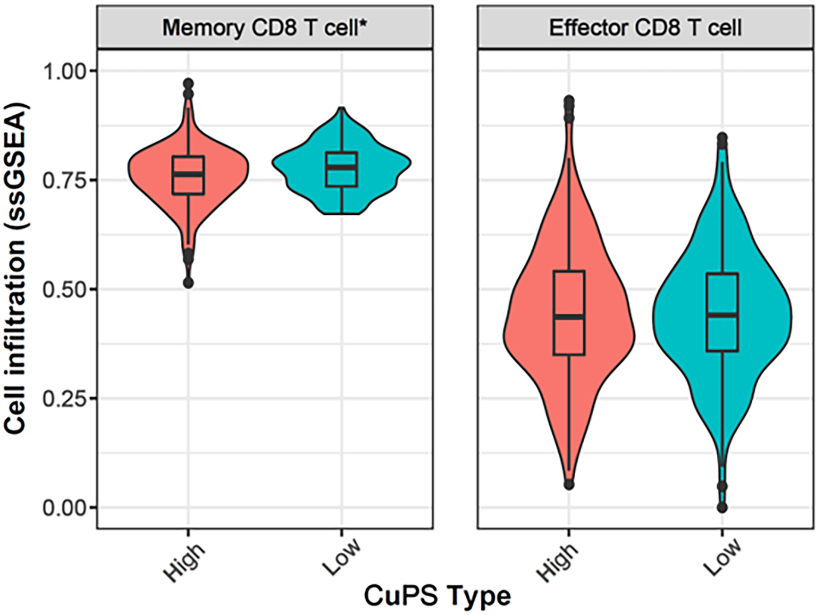


**Figure S5. Comparison of memory and effector CD8 T cells in the high vs low CuPS groups.** The abundance of memory and effector CD8 T cells were evaluated by ssGSEA algorithm. The violins in red presented high CuPS group, and the green presented low CuPS group.
